# A simple, high-throughput stabilization assay to test HIV-1 uncoating inhibitors

**DOI:** 10.1038/s41598-019-53483-w

**Published:** 2019-11-19

**Authors:** Alžběta Dostálková, Romana Hadravová, Filip Kaufman, Ivana Křížová, Kryštof Škach, Martin Flegel, Richard Hrabal, Tomáš Ruml, Michaela Rumlová

**Affiliations:** 1 Department of Biotechnology, University of Chemistry and Technology, Prague, 166 28 Czech Republic; 2 Institute of Organic Chemistry and Biochemistry IOCB Research Centre & Gilead Sciences, Academy of Sciences of the Czech Republic, Flemingovo nám. 2, 166 10 Prague, Czech Republic; 3 Department of Chemistry of Natural Compounds University of Chemistry and Technology, Prague, 166 28 Prague, Czech Republic; 4 NMR Laboratory, University of Chemistry and Technology, Prague, 166 28 Prague, Czech Republic; 5 Department of Biochemistry and Microbiology, University of Chemistry and Technology, Prague, 166 28 Prague, Czech Republic

**Keywords:** Retrovirus, High-throughput screening

## Abstract

Shortly after entering the cell, HIV-1 copies its genomic RNA into double-stranded DNA in a process known as reverse transcription. This process starts inside a core consisting of an enclosed lattice of capsid proteins that protect the viral RNA from cytosolic sensors and degradation pathways. To accomplish reverse transcription and integrate cDNA into the host cell genome, the capsid shell needs to be disassembled, or uncoated. Premature or delayed uncoating attenuates reverse transcription and blocks HIV-1 infectivity. Small molecules that bind to the capsid lattice of the HIV-1 core and either destabilize or stabilize its structure could thus function as effective HIV-1 inhibitors. To screen for such compounds, we modified our recently developed FAITH assay to allow direct assessment of the stability of *in vitro* preassembled HIV-1 capsid-nucleocapsid (CANC) tubular particles. This new assay is a high-throughput fluorescence method based on measuring the amount of nucleic acid released from CANC complexes under disassembly conditions. The amount of disassembled CANC particles and released nucleic acid is proportional to the fluorescence signal, from which the relative percentage of CANC stability can be calculated. We consider our assay a potentially powerful tool for *in vitro* screening for compounds that alter HIV disassembly.

## Introduction

To replicate successfully, viruses must deliver their genome into a host cell. During the extracellular phase of the viral life cycle, the viral genomic nucleic acid is protected by a protein shell formed by viral capsid (or nucleocapsid) proteins. Shortly after the virus enters the cell, this protective capsid shell is removed during a stepwise process called uncoating, in which the virus releases its genomic information. The basic steps of uncoating are regulated by many cellular and viral factors^1,2^. Generally, there are two distinct pathways of viral disassembly: the priming-based strategy and the compartment or milieu strategy^3^. The first is mediated by viral modifications resulting in metastability of the virus that allows disassembly of the protective shell in various cellular locations, depending on the virus. In the latter strategy, disassembly is triggered by changes in distinct subcellular compartments or environments. Some viruses employ a combination of these mechanisms, as well as assistance from various host cell factors^3^.

Disassembly of the mature capsid core, or uncoating, is not a well-understood post-entry event in the retroviral life cycle. The most comprehensive information is available for the HIV-1 core, which is composed of a protein shell formed by hexamers and pentamers of capsid protein. Intra- and inter-hexameric interactions provide well-balanced core stability, which plays a key role in the proper timing of uncoating. Any delay or acceleration in uncoating has a severe impact on retroviral replication. Mutations within the capsid domain (CA) that make the core either less or more stable impair HIV-1 infectivity. Not surprisingly, HIV-1 uncoating and other processes connected with early post-entry events depend on several cellular proteins, including the peptidyl-prolyl isomerase CypA^4^, the nuclear import receptor TNPO3^5,6^, the cleavage and polyadenylation specific factor 6 CPSF6^7,8^ and the nuclear pore proteins NUP153 and NUP358^9,10^. The precise mechanism, timing and site of HIV-1 uncoating have not yet been elucidated and the results gained so far are controversial (for review, see^11^). As host cell proteins recognize and bind to assembled rather than monomeric CA, it is very likely that the viral core persists largely intact upon entry the cytoplasm^12–14^. Among all, there are two leading models describing HIV-1 uncoating: the docking of intact core or its portion at the nuclear core^15–19^ and the second model where the HIV-1 core is uncoated in the cytoplasm^20–24^. In addition to cellular proteins, a small cofactor molecule, inositol hexakisphosphate (IP6), also may facilitate uncoating by affecting CA lattice stability^25–27^. However, the potential interplay between cellular protein and small molecule cofactors has not been determined.

The crucial role of uncoating in reverse transcription of retroviral genomic RNA makes it a suitable target for a new class of HIV-1 inhibitors. There are several available methods to monitor and quantify the stability and uncoating of HIV-1 core, including *in situ* uncoating assays to monitor uncoating within infected cells (for review, see^11,28,29^). The available *in vitro* methods use either authentic cores laboriously isolated from virions released from infected cells^13,20,30–32^ or those assembled *in vitro* from purified, recombinant CA or capsid-nucleocapsid (CANC) proteins^33,34^. *In vitro* assembled CANC tubular structures have a mature-like arrangement of CA subunits^35^ and can thus serve as surrogates for the HIV-1 core^34^. For example, in the capsid stabilization assay^33,34^, *in vitro* assembled CANC tubular structures are spontaneously disassembled upon incubation in a destabilization buffer and ultracentrifuged through a 70% sucrose cushion. The CA content in the pelleted fraction is compared to the input by Western blot.

Despite the availability of multiple methods to monitor the stability and uncoating of the HIV-1 core, there is no widely available, simple, high-throughput *in vitro* method to screen for compounds affecting the core stability. Here, we present a novel method to directly measure the kinetics of HIV-1 CA lattice destabilization. This assay is based on our recently developed protocol of the fast assembly inhibitor test for HIV (FAITH)^36^, which uses a purified HIV CANC protein that in the presence of a dually labelled TaqMan-based oligonucleotide (tqON) assembles into tubular structures. CANC assembly is initiated by interactions between the NC domain and tqON, which is then incorporated inside the assembling tubes and becomes protected. Subsequent addition of Exonuclease I degrades only free, non-incorporated tqON. Oligonucleotide cleavage leads to separation of the fluorescence label (FAM) from its quencher (BHQ). The fluorescence signal is proportional to the amount of non-assembled CANC particles. As this assay is suitable for a 96-well format, it can be used as a high-throughput screening method for compounds that interfere with HIV-1 assembly.

To screen for compounds that bind to the CA lattice, increase the core stability and block uncoating, we modified the FAITH assay to enable direct measurement of the kinetics of HIV-1 mature-like particle destabilization. Similar to FAITH, this new stability-monitoring assay is a high-throughput fluorescence method that measures the amount of nucleic acid released from preassembled CANC tubes. We optimized conditions that trigger destabilization of preassembled CANC tubes. Disassembly is followed by nuclease-mediated degradation of released dually labelled tqON, leading to release of the fluorophore from its quencher. The resulting fluorescence is proportional to the amount of disassembled CANC. In contrast to FAITH, this method is aimed at inhibitors that bind and stabilize the CANC array, and thus we named it disassembly inhibitor test for HIV (DITH).

In summary, we developed a new assay for high-throughput screening of compounds that by binding to CA stabilize the viral hexameric lattice and potentially function as uncoating inhibitors.

## Results

### Optimization of HIV-1 CANC disassembly

**Figure 1:**
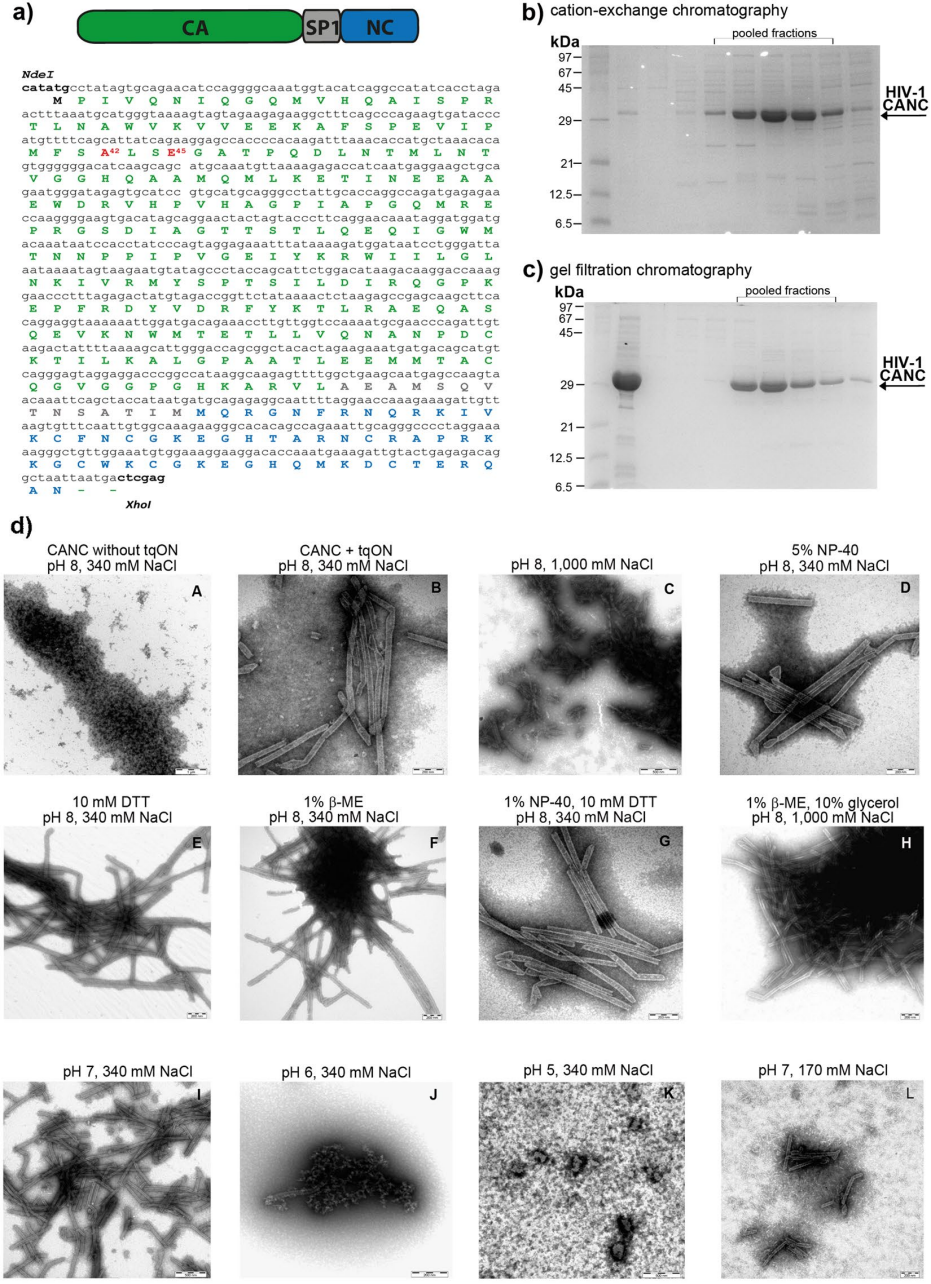
Purification, assembly and disassembly of HIV-1 CANC. (**a**) Schematic representation of HIV-1 CANC protein and its full-length amino acid sequence; mutations used in this study are shown in red. (**b,c**) Coomassie Brilliant Blue -stained SDS polyacrylamide gels showing pooled fractions of HIV-1 CANC protein after purification by (**b**) cation-exchange and (**c**) gel filtration chromatography. (**d**) TEM analysis of negatively stained HIV-1 CANC protein under assembly/disassembly conditions. HIV-1 CANC protein was assembled in the absence (A) or presence (B–L) of tqON in assembly buffer containing 50 mM Tris, pH 8, 340 mM NaCl and 1 µM ZnCl_2_. The indicated reactant (C–L) was added to the assembled tubular structures, and the incubation proceeded overnight at laboratory temperature.

To establish an assay for quantification of HIV-1 CANC array destabilization, we sought to determine conditions required for transition of compact *in vitro* assembled CANC-tqON tubes into oligo- or monomeric protein subunits with tqON accessible for Exonuclease I degradation. HIV-1 CANC protein, consisting of full-length CA, SP1 and NC sequence (Fig. 1a) was purified by combination of cation-exchange and gel filtration chromatography (Fig. 1b,c) as described in Material and methods and elsewhere^36–38^. The tubular structures from the purified HIV-1 CANC protein were first assembled as previously described^36^ in the presence of tqON under the following reaction conditions: 18 µM CANC, 2.19 µM tqON, 50 mM Tris, pH 8, 340 mM NaCl and 1 µM ZnCl_2_. To destabilize these *in vitro* assembled tubular structures (shown in Fig. 1d, panel B), the composition of the buffer was modified by addition of one or more of the following components: NaCl (340 mM to 1 M), the detergent NP-40 (0.5–5%), and the reducing agents DTT (1–10 mM) or β-ME (0.1–1%). Following overnight incubation at room temperature with gentle shaking (480 rpm), the samples were negatively stained and analyzed by TEM (Fig. 1d, panels C–H). None of these conditions led to significant destabilization of the assembled CANC structures. Next, we attempted to destabilize the CANC arrays either by lowering the pH of the final reaction buffer (in the range from pH 5–7) (Fig. 1d, panels I–K) or by lowering both the pH and ionic strength to a final pH of 7 and NaCl concentration of 170 mM (Fig. 1d, panel L). Both conditions led to destabilization of the CANC arrays and caused partial or nearly complete disassembly of the CANC tubular structures (Fig. 1d, panels J–L).

**Figure 2:**
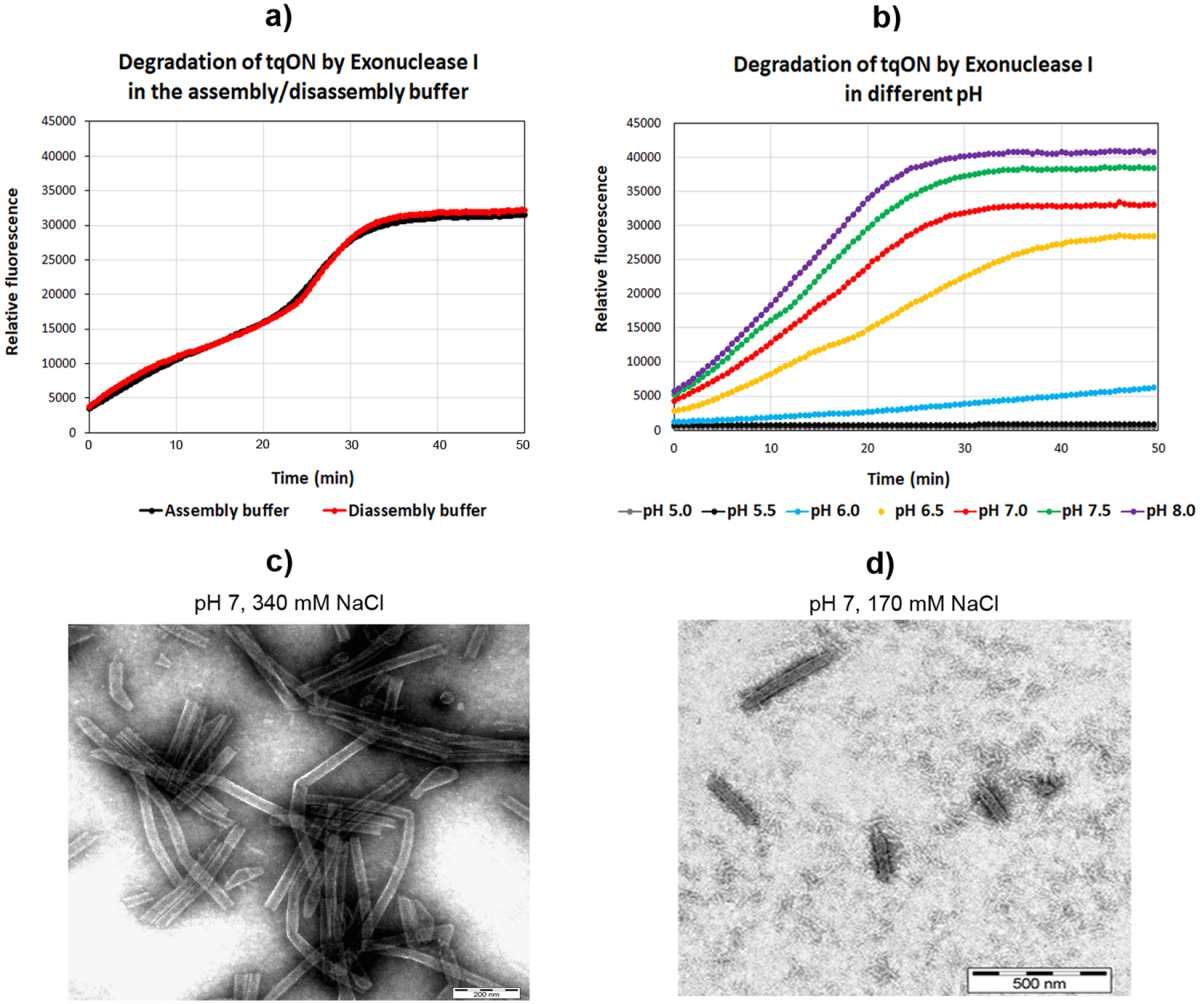
Exonuclease activity and TEM analysis of CANC particles under assembly/disassembly conditions. Digestion of FAM-tqON by Exonuclease I in buffers with (**a**) different NaCl concentration and (**b**) pH. (**c**) TEM image of CANC assembled in presence of tqON in the assembly buffer (50 mM Tris, pH 7, 340 mM NaCl, 1 µM ZnCl_2_). (**d**) TEM image of the CANC particles shown in (**c**) after incubation in the disassembly buffer (50 mM Tris, pH 7, 170 mM NaCl, 1 µM ZnCl_2_).

Next, we analyzed whether the conditions triggering CANC disassembly were also suitable for Exonuclease I activity, which is an essential part of the assay. The lower ionic strength (170 mM NaCl) did not influence tqON degradation, and the activity of the exonuclease was the same at 340 mM NaCl as at 170 mM NaCl (Fig. 2a). However, exonuclease activity was strongly inhibited at pH values under 7 (Fig. 2b). To maintain the Exonuclease I activity, we adjusted the pH of the assembly buffer to 7 and the ionic strength to 340 mM NaCl. Under these conditions, CANC assembled into stable, regularly ordered uniform tubular structures (Fig. 2c). Destabilization was triggered by dilution of these assembled CANC tubes with an equal volume of 50 mM Tris, pH 7, 1 µM ZnCl_2_ to yield the following final composition of disassembly buffer: 50 mM Tris, pH 7, 170 mM NaCl, 1 µM ZnCl_2_ (Fig. 2d).

### Verification of the DITH principle

The CA domain within the CANC protein is ordered in a mature-like arrangement and therefore can serve as a surrogate for the HIV-1 core, despite the presence of the NC domain^34,35^. As our DITH assay is dependent on the accessibility of tqON to Exonuclease I, we analyzed whether the change in ionic strength used to trigger CANC disassembly affects the interaction of the oligonucleotide with the NC domain. To do so, we performed fluorescence anisotropy under non-assembly conditions (i.e., at low protein concentration ranging from 1–6 µM) (Fig. S1). As exonuclease was not added to the reaction, we used single-labelled FAM-ON rather than double-labelled ON. As expected, the binding of FAM-ON to CANC was stronger (Kd = 0.7 µM) at the low ionic strength of the disassembly buffer (50 mM Tris, pH 7, 170 mM NaCl, 1 µM ZnCl_2_) than in the assembly buffer (50 mM Tris, pH 7, 340 mM NaCl, 1 µM ZnCl_2_) (Kd = 4.5 µM). This result confirmed that the disassembly conditions do not facilitate release or dissociation of tqON from NC. On the contrary, the binding of tqON to NC was even stronger under low ionic strength conditions, and thus disassembly was achieved mainly by affecting the CA-CA interactions. This destabilization of the CA lattice subsequently led to exposure of tqON, making it available for exonuclease cleavage. Despite the different binding affinities at different ionic strengths, the result of tqON exonuclease degradation remained unchanged. This confirms that merely binding to non-assembled CANC does not protect tqON from degradation and suggests the robustness of our method to measure the proportion of assembled versus non-assembled CANC.

**Figure 3:**
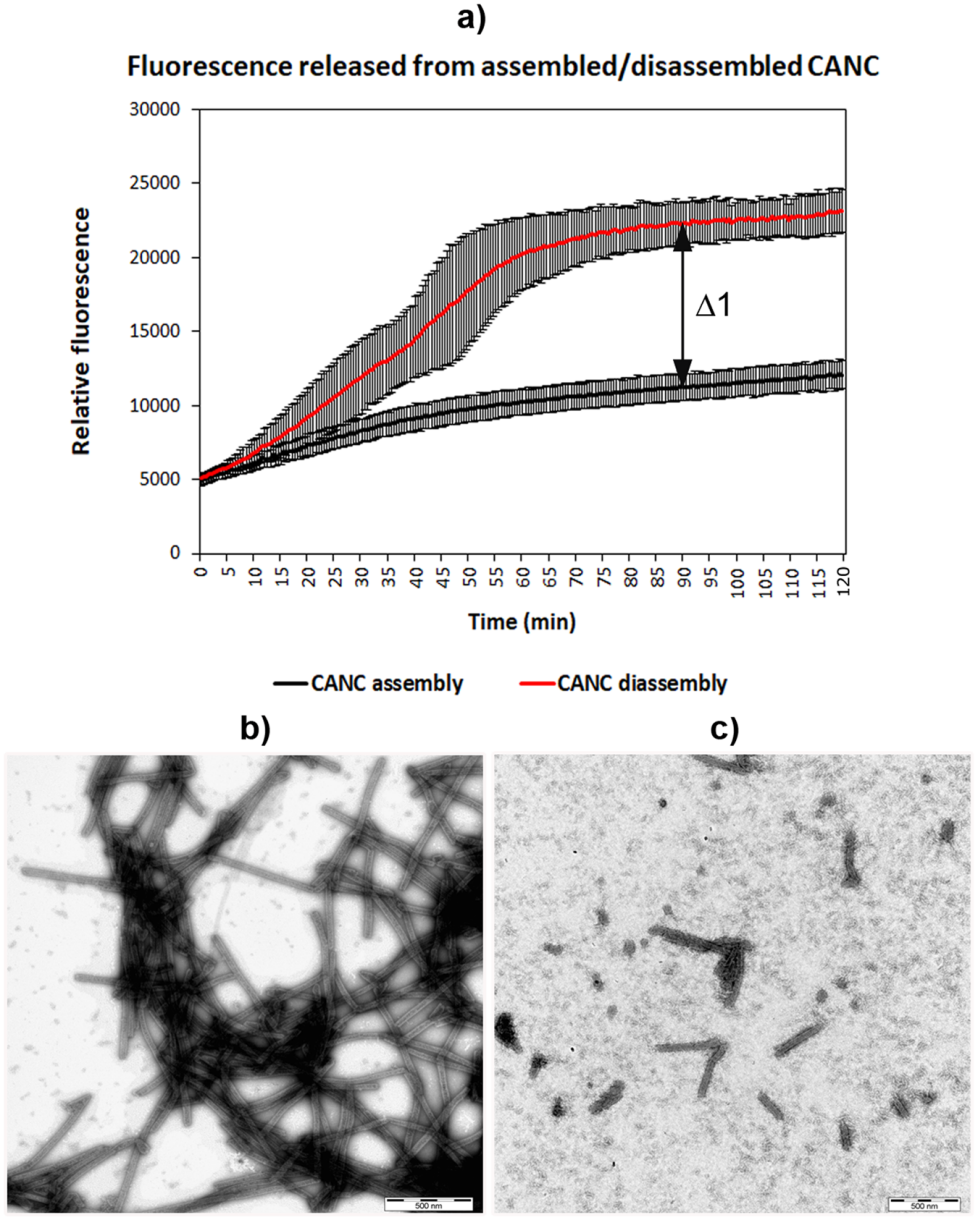
CANC disassembly monitored by DITH and TEM. (**a**) Fluorescence emission curves demonstrating the kinetics of tqON release from preassembled CANC particles incubated in assembly buffer (black curve) and disassembly buffer (red curve); the vertical bars represent standard deviations for single measured points. The relative stabilization was then calculated as the difference between the relative fluorescence of tqON after 90 min in the disassembly and assembly reactions (Δ1 in panel a). The stability of wt in assembly buffer was considered as 100%, the stability in disassembly buffer as 0%. (**b,c**)TEM analysis of negatively stained HIV-1 CANC protein preassembled in the presence of tqON in assembly buffer and then incubated in (**b**) assembly buffer or (**c**) disassembly buffer.

To quantify the HIV-1 CANC tube disassembly, we compared the fluorescence of the fluorophore (FAM) released from the tqON-CANC tubes in disassembly buffer with that released from the tubes in assembly buffer upon addition of Exonuclease I. As the destabilization is triggered by dilution of the assembly buffer, we wanted to rule out the possibility that disassembly resulted from merely lowering the CANC protein concentration. Following the *in vitro* assembly of tqON-CANC tubular structures in the pH 7 buffer, the sample was divided in two aliquots that were diluted into different buffers to yield the same final protein and tqON concentrations. One aliquot was diluted into the assembly buffer, while the second one was diluted into 50 mM Tris, pH 7, 1 µM ZnCl_2_. Both samples were then incubated overnight with gentle agitation at laboratory temperature. The next day, Exonuclease I was added to both samples and an increase in fluorescence was monitored for 120 min (Fig. 3a). The samples were negatively stained and analyzed by TEM (Fig. 3b,c). The fluorescence increase confirmed that the preassembled CANC particles incubated at the lower ionic strength of the disassembly buffer were more prone to tqON degradation than those incubated in the assembly buffer (Fig. 3a, red and black lines, respectively). In accord with this, we observed typical tubular structures in the sample diluted into assembly buffer (Fig. 3b), while fully or partially disassembled tubes were observed in the sample diluted into disassembly buffer (Fig. 3c). The relative stabilization of wt CANC (Δ1) was then calculated from the difference between the fluorescence of released and degraded tqON at 90 min in the disassembly and assembly reactions (Fig. 3a).

### Validation of DITH using HIV-1 CA mutants with altered core stability

**Figure 4:**
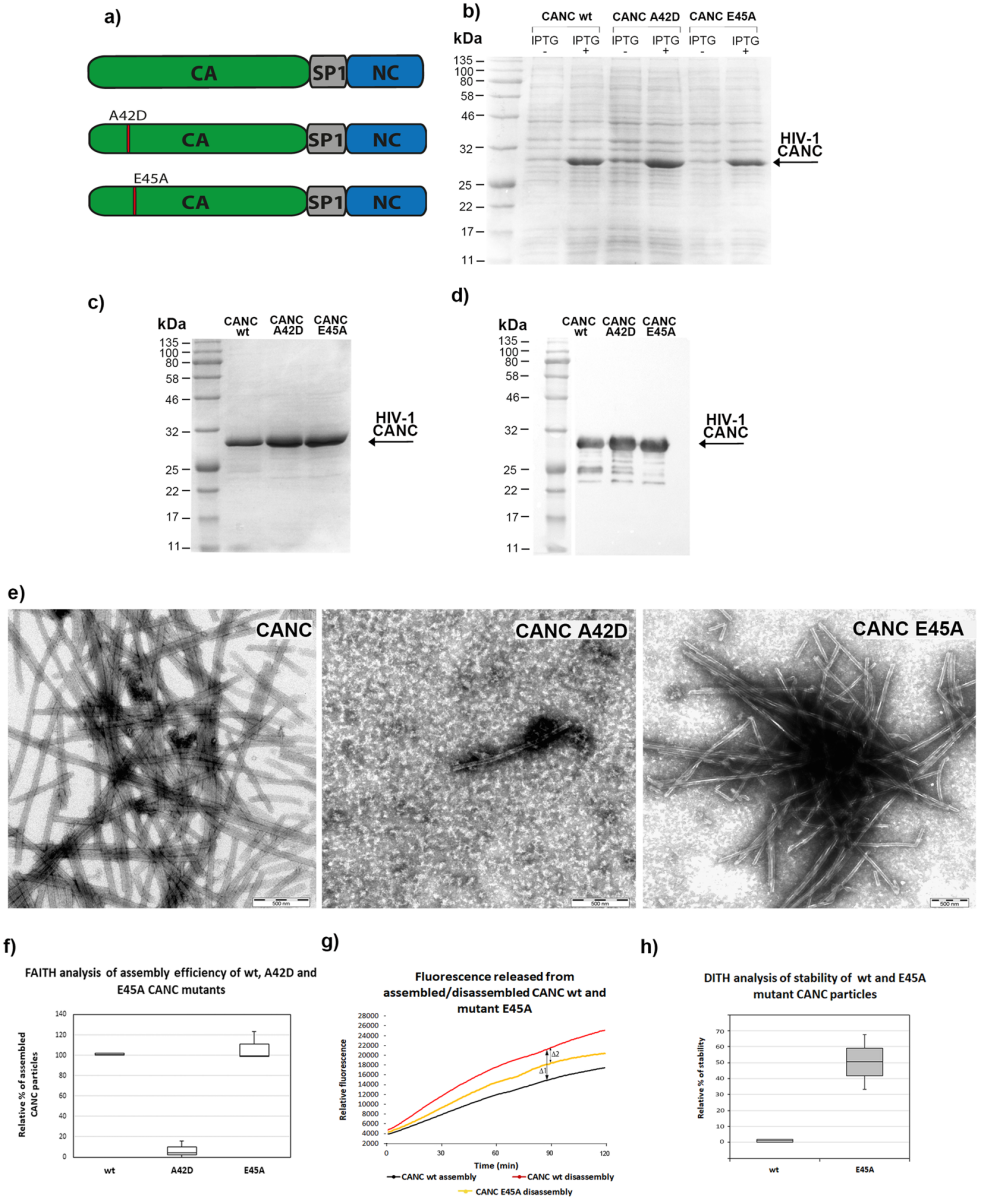
Expression and purification of HIV-1 A42D and E45A CANC mutants and assembly/disassembly analysis. (**a**) Schematic representation of the A42D and E45A mutations introduced within the CA domain of HIV-1 CANC. (**b**) Coomassie Brilliant Blue-stained SDS polyacrylamide gels showing the expression of wt HIV-1 CANC and the A42D and E45A CA mutants. (**c**) Coomassie Brilliant Blue-stained SDS polyacrylamide gel and (**d**) Western blot analysis of purified wt HIV-1 CANC and the A42D and E45A CA mutants. Colour prestained protein marker (NEB) was visualized by a digital camera in bright field (Fig. S2a), HIV-1 CANC proteins in chemiluminescent mode (Fig. S2b). (**e**) TEM analysis of negatively stained wt HIV-1 CANC and the A42D and E45A CA mutants assembled in the presence of tqON in assembly buffer. (**f**) FAITH quantification of wt HIV-1 CANC and CA mutant assembly in the presence of tqON. (**g**) Fluorescence emission curves demonstrating the kinetics of tqON release from preassembled wt and E45A CANC particles incubated in assembly buffer (black curve) and disassembly buffer (red and yellow curves, respectively). The relative stabilization was calculated as the difference between the relative fluorescence of tqON at 90 min in the disassembly and assembly reactions according the calculation: relative percent of stabilization = 100 * Δ2/Δ1. (**h**) DITH quantification of the relative stability of preassembled wt and E45A CANC particles incubated in the disassembly buffer measured and calculated as described in (**g**). Relative stability of wild type in disassembly buffer was considered as 0%.

To validate DITH, we used two HIV-1 CA mutants with distinct core stability: the unstable A42D mutant and the hyperstable E45A mutant^30,39^ (Figs. 1a, 4a). Using EMILI mutagenesis^40^ we introduced the point mutations in CANC pET22b vector, and following their expression (Fig. 4b), we optimized purification of both CANC mutant proteins (Fig. 4c,d) using the same work-flow described for wild type. Next, we tested the assembly efficiency of these mutants using TEM and the FAITH assay (Fig. 4e,f). We confirmed that the wild-type (wt) and E45A CANC proteins assembled efficiently into long tubular structures. In contrast, the ability of the A42D mutant to assemble was impaired. Therefore, the DITH assay was performed only with the hyperstable E45A CANC mutant. Preassembled wt and E45A CANC particles were incubated overnight in the assembly and disassembly buffers, and fluorescence was measured after Exonuclease I addition (Fig. 4g). Fluorescence released from the wt or E45A CANC in assembly buffer is shown in black, while fluorescence released from the wt and E45A in disassembly buffer is shown in red and yellow (Fig. 4g), respectively. The relative stabilization for wt and E45A mutant tubes was calculated as the difference between the fluorescence of degraded tqON at 90 min in the disassembly and assembly reactions for the wt (Δ1 Fig. 4g) and E45A mutant (Δ2 Fig. 4g). The relative percent of stabilization was then determined using simple calculation: relative % of stability = 100 * Δ2/Δ1. The calculated relative percentage values of stability were then plotted and compared with the wild type in the disassembly buffer whose stability was considered as 0% (Fig. 4h). As expected, E45A CANC mutation increased stability of CANC tubes in the disassembly buffer by about 50% compared to the wild type (Fig. 4h).

### Evaluation of DITH with the capsid inhibitor PF74

As the main purpose of our *in vitro* disassembly method is to screen for compounds and small molecules that alter the stability of the hexameric array of CANC tubular structures, we used the inhibitor PF74 for further DITH validation. PF74 is a small molecule that binds to the capsid core and inhibits HIV-1 at an early stage of infection^41^. PF74 preferentially binds to the hexameric lattice of the capsid core, affecting both intra- and inter-CA hexamer interactions^14,42,43^. This alteration subsequently results in enhanced stability of the HIV-1 capsid core, which in turn affects reverse transcription and viral infectivity.

**Figure 5:**
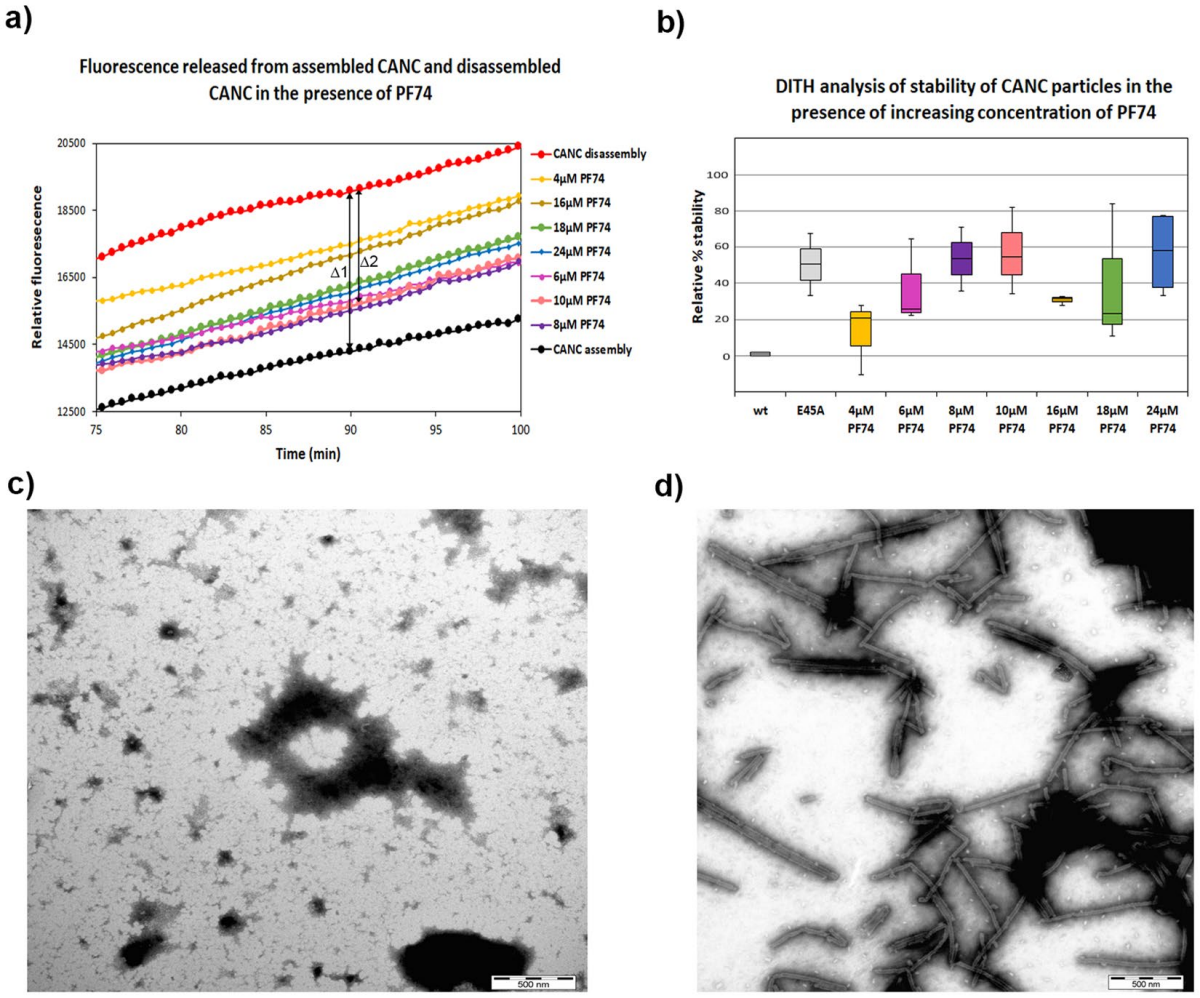
DITH and TEM analysis of the effect of PF74 on the stability of HIV-1 CANC. (**a)** An enlargement of a graph (Fig. S3) showing fluorescence emission curves demonstrating the kinetics of tqON release from preassembled wt CANC particles incubated in assembly buffer (black curve) and disassembly buffer (red curve) in the absence of PF74 or in the disassembly buffer at increasing concentration of PF74: yellow (4 µM), pink (6 µM), violet (8 µM), salmon (10 µM), brown (16 µM), green (18 µM) and blue (24 µM). The stabilization effect of PF74 was calculated as the difference between the relative fluorescence of tqON at 90 min in the disassembly and assembly reactions according the calculation: relative percent of stabilization = 100*Δ2/Δ1. (**b**) DITH quantification of the relative stability of preassembled wt particles incubated in the disassembly buffer in the presence of increasing amount of PF74, measured and calculated as described in (**a**). Relative stability of wild type in disassembly buffer is considered as 0%. Relative percent of E45A CANC mutant stability in the disassembly buffer, measured and calculated as it is shown in 4 g, was added. (**b,c**) Following DITH, an untreated sample (**b**) and the sample containing 18 µM PF74 (**c**) were negatively stained and analyzed by TEM.

To evaluate whether optimized conditions for destabilization of the CA hexameric lattice of preassembled CANC tubes would allow measurement of the stabilizing effect of bound small molecules, we used DITH to quantify the stabilization effect of PF74 binding to CANC. Various concentrations (4 to 24 µM) of PF74 were added to the assembled CANC tubes following 1 h incubation. The samples were then divided into two aliquots and diluted into either assembly or disassembly buffer. Following overnight incubation, Exonuclease I was added to the samples and the kinetics of fluorescence release were measured (Figs. 5a, S3). Fluorescence released from the wt assemblies in assembly buffer is shown in black (Fig. 5a), while fluorescence released from the wt in disassembly buffer is shown in red (Fig. 5b). Fluorescence released from the PF74 treated wt in disassembly buffer is shown in yellow (4 µM PF74), pink (6 µM PF74), violet (8 µM PF74), salmon (10 µM PF74), brown (16 µM PF74), green (18 µM PF74) and blue (24 µM PF74). The proportion of assembled and degraded wt tubes (Δ1) was calculated as the difference between the fluorescence of tqON at 90 min in the disassembly and assembly reactions (Fig. 5a). This fluorescence range was then used to calculate the relative stabilization of CANC assemblies by PF74 as the difference between the fluorescence of released tqON from PF74 treated tubes and the wt upon 90 min incubation in the disassembly buffer (e.g. the difference for 10 µM PF74 is shown as Δ2 in Fig. 5a). The relative percent of stabilization was then calculated as follow: relative percentage of stability = 100 * Δ2/Δ1. The relative percentage of stability of wt CANC assemblies for each tested PF74 concentration was then plotted and compared with the wild type in the disassembly buffer whose stability was considered as 0% (Fig. 5b). Based on the DITH results, we observed a PF74 induced increase of CANC stability in comparison to untreated sample in the whole range of tested PF74 concentrations (Fig. 5b). Lower stabilization was observed at PF74 concentrations ranging from 16 to 18 µM, where we consistently observed a slight decrease in stabilization capacity of PF74, which was overcome at higher PF74 concentration (24 µM) (Fig. 5b). Stabilization effects of E45A mutation (Fig. 5b, grey) was similar to that of PF74 at concentrations ranging from 8–10 µM (Fig. 5b violet and salmon, respectively).

Following DITH, sample treated with 10 µM PF74 and untreated CANC sample were negatively stained and analyzed by using TEM (Fig. 5c,d). The TEM analysis clearly confirmed that the addition of PF74 stabilized the CANC tubular structures and prevented their degradation during incubation in disassembly buffer (Fig. 5d). In contrast, in the absence of PF74, the capsid lattice of CANC tubular structures disintegrated (Fig. 5c).

### Effect of inositol hexakisphosphate (IP6) on HIV-1 CANC stability

**Figure 6:**
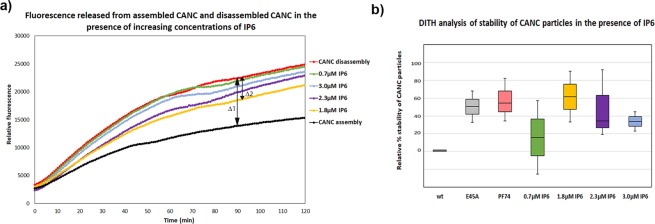
Effect of IP6 on the relative stability of CANC HIV-1 particles compared to effect of E45A mutant and PF74 determined by DITH. Tubular particles were assembled from HIV-1 CANC in the presence of tqON and indicated concentrations of IP6 in assembly buffer. Samples were incubated for 3 h and then diluted into assembly or disassembly buffers and incubated overnight. (**a**) Fluorescence emission curves demonstrating the kinetics of tqON release from preassembled wt CANC particles incubated in assembly buffer (black curve) and disassembly buffer (red curve). The relative stabilization for various IP6 concentrations (from 0.7–3.0 µM) was calculated as the difference between the relative fluorescence of tqON at 90 min in disassembly and assembly reactions according the calculation: relative percent of stabilization = 100 * Δ2/Δ1. (**b**) DITH quantification of the relative percent of stability of preassembled wt particles incubated in disassembly buffer in the presence of increasing amount of IP6, measured and calculated as described in (**a**). Relative percent of E45A CANC mutant stability and wt CANC in the presence of 10 µM PF74 in the disassembly buffer is also shown.

In addition to synthetic organic compounds such as PF74, small cellular polyanions such as IP6 can be incorporated into assembling immature HIV-1 particles and enhance core stability^26^. We employed the DITH assay to test the stabilization effect of IP6 on assembled mature-like CANC tubular structures. The tubular particles were preassembled from HIV-1 CANC protein in assembly buffer in the presence of tqON and various concentrations of IP6: 0.7 µM (green). Following 3 h incubation, the samples were divided into two aliquots and diluted into either assembly buffer or disassembly buffer. After overnight incubation, Exonuclease I was added to all the samples and the kinetics of fluorescence release were measured (Fig. 6a). Fluorescence released from the wt assemblies in assembly and disassembly buffers is shown in black and red, respectively (Fig. 6a). Fluorescence released from the wt in disassembly buffer at various IP6 concentrations is shown in green (0.7 µM), yellow (1.8 µM), violet (2.3 µM) and blue (3 µM) (Fig. 6a).

Relative percentage of stabilization effect of IP6 was calculated as described above for PF74, plotted and compared with the wild type whose stability in the disassembly buffer was considered as 0% (Fig. 6b). The greatest stabilizing effect (by 1.5-fold) was observed for CANC:IP6 ratios ranging from 1:10 to 1:8, corresponding to IP6 concentrations of 1.8 µM to 2.3 µM, respectively. A comparison of relative stability of E45A mutant (Fig. 6b, grey) and wt CANC in the presence of 10 µM PF74 (Fig. 6b, salmon) in the disassembly buffer revealed similar level of stabilization as that observed for 1.8 µM IP6 (Fig. 6b, yellow).

## Discussion

Here, we describe development of a new high-throughput fluorescence method that enables direct measurement of the kinetics of disassembly of HIV-1 CANC tubular particles formed *in vitro*. The method is based on measurement of fluorescence generated upon degradation of an oligonucleotide that becomes accessible upon disassembly of CANC tubular structures. The oligonucleotide is a dually labelled, TaqMan-based, 40-base-pair, single-stranded DNA called tqON. When tqON is incorporated inside the preassembled CANC tubular structures, it is protected from degradation by Exonuclease I. Uncoating or disassembly of the hexameric CA lattice of CANC complexes is triggered by dilution of the preassembled CANC tubular structures into disassembly buffer. The disintegration of the tubular structures renders the released tqON vulnerable to degradation by Exonuclease I. Exonuclease cleavage leads to separation of the fluorescence label (FAM) from the quencher (BHQ). The amount of disassembled CANC particles is therefore proportional to the FAM-derived fluorescence signal, from which the relative percentage of CANC destabilization or stabilization can be calculated.

HIV-1 CANC can assemble *in vitro* into narrow tubular structures^38,44^. Despite the presence of the NC domain, the CA domain of CANC adopts a mature-like arrangement^35^. Therefore, this *in vitro* system can be used experimentally as a surrogate for the HIV-1 core^34^, as demonstrated with the capsid stabilization assay^33,34^. In this assay, CANC protein is assembled *in vitro* in the presence of a 25-nucleotide DNA strand (TG_25_). The *in vitro* preassembled CANC tubular structures are incubated in a destabilization buffer to instigate disassembly, and the material is ultracentrifuged through a 70% sucrose cushion. The level of CANC stability is determined by comparing the CA content in the pelleted fraction with the input CA amount using Western blot^33^. Our new DITH method uses the same HIV-1 CANC protein, but differs in its usage of dually labelled FAM-BHQ oligonucleotide. This arrangement is advantageous because the amount of disassembled CANC particles is proportional to the FAM-derived fluorescence signal. Thus, DITH allows direct real-time determination of stabilization or destabilization without the need for ultracentrifugation and Western blotting steps. As DITH can be carried out entirely in a 96-well plate, it is optimal for small molecule screening or testing of CA-binding factors or proteins.

During optimization of disassembly of the CANC tubular structure, we assessed various conditions that led to the decomposition of CANC complexes and exposure of tqON to Exonuclease I cleavage. The most efficient disassembly was achieved by lowering the pH below pH 6. However, this non-physiological acidity could affect the overall or local charge of the CA and NC domains or the compounds tested, and thus alter their interactions. Moreover, we found that low pH conditions were not suitable for the assay, as the exonuclease was inhibited. However, we found that CANC tubular structures assembled at pH 7 were more prone to disassembly that those assembled at pH 8. Therefore, we altered conditions for the *in vitro* assembly of CANC tubular structures and preassembled the particles at pH 7. Disassembly was then triggered by simply diluting the preassembled CANC particles into the same pH 7 buffer with a lower ionic strength to yield a final NaCl concentration of 170 mM. The change in ionic strength had no effect on Exonuclease I activity.

We used several means to validate and evaluate the DITH method. First, we prepared CANC protein with E45A mutation in the CA domain, which has been shown to form a hyperstable core^30,39^. In accord with previous data, our results showed that the E45A mutant exhibited 50% higher stability than the wild-type. We also used DITH to assess the core stabilization effect of PF74, a small molecule that binds the CA hexameric lattice^14,41–43^. Results of previous studies on effects of PF74 are conflicting. *In vitro* experiments demonstrated completely converse impact of PF74 on HIV-1 core: it either accelerated HIV-1 core disassembly by reducing its stability^41–43^ or it increased the stability of HIV-1 CA cores thus slowing down the disassembly^42,45^. Recently, researchers using atomic force microscopy (AFM) measured stiffness of capsids assembled *in vitro* and HIV-1 isolated cores. They showed that binding of PF74 increased the stability of both structures in a concentration-dependent manner^46^. We obtained similar results by using DITH, finding that the CANC stability was enhanced in the presence of PF74. However, even though we observed a concentration-dependent effect of PF74 on HIV-1 CANC stabilization in the inhibitor concentration ranging from 4–10 µM, we consistently observed a slight drop in PF74 stabilizing effect at concentrations within 16–18 µM. This effect disappeared at PF74 concentration 24 µM, and the same level of stabilization, observed for 10 µM was re-established. Due to DMSO concentration limitation (maximum in the reaction is 1%), we could not increase the PF74 concentration above 24 µM, which prevented to determine further trend of stabilization. However, based on the data published by Rankovic *et al*., who showed the CA assemblies stability increases in the PF74 concentration-dependent manner, but not above 20 µM PF 74^46^, we can hypothesize that there would not be any significant increment in CANC stability at PF74 concentration higher than 24 µM. The reason for the observed drop in PF74 stabilization at 16–18 µM however, remains unclear. Nevertheless, our data on the comparison of the stabilization effect of PF74 and E45A mutation are in concert with those showed by Rankovic *et al*.^46^. Using AFM they showed that the stiffness, observed upon binding of PF74 at 10 µM concentration, of *in vitro* assembled capsids achieved the level similar to that of the E45A mutant^46^. In our assay, similar level of stabilization observed for E45A mutant was achieved at PF74 concentration of 8 µM.

Our previous work indicated that PF74 does not affect the *in vitro* assembly of either mature-like or immature-like structures^36^, in accord with a published crystal structure that reveals a binding pocket between helices 3 and 4 of HIV-1 NTD-CA^41^. Other *in vitro* studies have suggested that PF74 binding significantly increases the rate of CA assembly^41,42^. We observed previously that PF74 affects the structure of immature-like particles, which form mature-like short rods instead of spherical particles^36^, suggesting that the binding of PF74 may shift the equilibrium towards mature-like assembly^36^. This is in agreement with the results on core stabilization described in the present work.

Additionally, we used DITH to evaluate the effect of small cellular polyanion IP6, which can stabilize immature HIV-1 capsid lattices by binding to two lysine residues in CA and SP1 regions of Gag hexamers^26^. The arginine residue at the N-terminus of CA, which becomes unmasked by proteolytic cleavage of Gag during maturation can serve as an alternative IP6 binding site^26,27^. However, quantitative analysis of stabilizing effect of IP6 on mature CA lattice was not published. Here, using DITH, we tested concentration-dependent effect of IP6 on stabilization of CANC mature-like structures and we identified the optimal ratio to achieve the highest stability. We found that 1.8 µM IP6 provides similar 50% increment of CANC tubular structures stability as that shown for E45A mutant, 8–10 µM PF74.

These data clearly show that DITH is a reliable method that can be used for initial screening of compounds that bind to and stabilize the CA lattice and have the potential to block HIV-1 infectivity. The method might also be applicable to compare the effects of a newly designed series of inhibitors that potentially form more extensive contacts with CA, as recently proposed by molecular docking and structural analysis for molecules GS-CA1 and GS-6207^47^.

## Material and Methods

### Cloning and mutagenesis

HIV-1 CANC, encoding CA, SP1 and NC fusion protein was prepared as described earlier^36,37^. Two point mutations A42D and E45A were prepared by EMILI mutagenesis^40^, using following primers: 5′ A42D CCATGTTTTCAGATCTATCAGAAGGAGCCACCCCACAA, 3′A42D CCTTCTGATAGATCTGAAAACATGGGTATCACTTCTGGGC, 5′E45A CAGCATTATCCGCGGGAGCCACCCCA CAAGATTTAAAC, 3′E45A GGGGTGGCTCCCGCGGATAATGCTGAAAACATGGGTATCAC. Newly created vectors were verified by sequencing.

### Expression and purification

The HIV-1 CANC wt and A42D and E45A mutant proteins were purified as previously described^36–38^ with some modifications. The HIV-1 CANC proteins were expressed in *E. coli* BL21 (DE3). The bacterial pellet (5 g) was resuspended in 25 ml of buffer D (20 mM Tris-HCl, pH 8, 0.5 M NaCl, 10% glycerol, 1 mM EDTA, 10 mM DTT, 1 mM Triton X-100, 1x Health Protease Inhibitor Cocktail) and the cells were disrupted by sonication (4 × 20 s) on ice. Polyethyleneimine was added to the cell lysate to a final concentration of 0.15% (w/v), and cell debris and nucleic acids were removed by ultracentrifugation (Beckman, TI 90, 55,000 rpm, 3 h, 4 °C). HIV-1 CANC protein was diluted to final NaCl concentration of 100 mM with buffer E (20 mM Tris-HCl, pH 8.0, 50 µM ZnCl_2_, 10 mM DTT, 1x Health Protease Inhibitor Cocktail), filtrated through a 0.45 µm pore filter (Amicon) and loaded onto a HiPrep™SP FF 16/10 column (GE Healthcare) equilibrated in buffer E. The bound proteins were eluted with a NaCl gradient from 0.1 M to 1 M NaCl in buffer E. The fractions containing CANC protein were pooled and concentrated to an approximate volume of 5 ml using an Amicon®Ultra-4 filter. The sample was then loaded onto a HiLoad™26/600 Superdex™ column equilibrated in buffer E with 0.5 M NaCl. The HIV-1 CANC protein from pooled fractions was concentrated to 2–4 mg/ml and stored at −80 °C. The purity of the protein was analyzed by SDS-PAGE and verified by Western blot using anti-HIV-1 CA antibodies produced in house^48^.

### Fast assembly inhibitor test for HIV (FAITH)

FAITH was used for *in vitro* testing of inhibitors of assembly of HIV-1 particles^36^. A 60 µg portion of HIV-1 CANC was pre-incubated with inhibitor for 1 h on ice. Following incubation with 3 µg of dually labelled oligonucleotide (tqON) in assembly buffer (50 mM Tris, pH 8.0, 1 µM ZnCl_2_, 340 mM NaCl) in a 96-well for 3 h at room temperature, Exonuclease I (ExoI) with Mg^2+^ ions was added to the mixture. The fluorescence of the fluorophore released from degraded tqON present in the solution was measured using a Tecan M200Pro plate reader.

### Stabilization fast assembly inhibitor test for HIV (DITH)

Small molecules were added at a final concentration of 18 µM to HIV-1 CANC tubular structures in a 96-well plate. The mixture was incubated for 3 h at room temperature. Next, 100 µl of disassembly buffer (50 mM Tris, pH 7.0, 1 µM ZnCl_2_) was added to the each sample in the plate, and incubation under moderate agitation continued overnight at room temperature. ExoI and Mg^2+^ ions were added 16 h later, and fluorescence was measured using a Tecan M200Pro plate reader. The relative stabilization for wt and E45A mutant, PF74 or IP6 treated CANC assemblies was calculated as the difference between the fluorescence of degraded tqON at 90 min in disassembly and assembly reactions for the wt (Δ1) and E45A mutant, PF74 and IP6 treated CANC assemblies (Δ2). The relative percent of stabilization was then determined using following formula: relative percentage of stability = 100 * Δ2/Δ1. The calculated relative percentage values of stability were then plotted and compared with the wild type in disassembly buffer whose stability was considered as 0%

### Fluorescence anisotropy binding assay

The fluorescence anisotropy binding assay was carried out as previously described^49^. Briefly, a solution of 10 nM 5′-FAM-labeled ssDNA (5′- GATTAAAAGTGAAAGTAAACTC-3′) was titrated with HIV-1 CANC to an overall protein concentration ranging from 8 to 6,000 nM. Changes in 5′-FAM ON DNA fluorescence anisotropy induced by changes in the CANC protein concentration and the equilibrium dissociation constant (Kd) were determined as previously described^50^.

### Synthesis of PF74 derivatives

The PF74 molecule consists of three synthons: 2-(2-methyl-1H-indol-3-yl)acetic acid, L-phenylalanine and N-methylaniline. The compound was purified by flash column chromatography on silica gel and analytically characterized by NMR and mass spectrometry. The thin-layer chromatography (TLC) was performed using (TLC Silica gel 60 F254, Merck). TLC detection (254 nm). The column chromatography was performed using silica gel (100–160 μm, Merck) and glass column with 2 cm in diameter and 12 cm in height. NMR spectra were acquired using device from Agilent Technologies with working frequency 400 MHz, chemical shift in ppm (δ) and J-constants in Hz. The mass spectra were measured using LC-MS TSQ Quantum Access Max (Thermo). The products were identified via 1 H NMR, 13C NMR, COSY, HMQC and MS (Supplementary Table 1).

All data generated or analyzed during this study are included in this published article. Data that are not shown are available from the corresponding author on reasonable request.

## Supplementary information

Supplementary data
