# Supplementary material for: A simple, high-throughput stabilization assay to test HIV-1 uncoating inhibitors
Source: Sci Rep. 2019 Nov 19;9:17076. doi: 10.1038/s41598-019-53483-w (PMC6863892; doi:10.1038/s41598-019-53483-w)
Supplement: Supplementary file 1 — Supplementary data [file 41598_2019_53483_MOESM1_ESM.docx]

**A simple, high-throughput stabilization assay to test HIV-1 uncoating inhibitors**

Alžběta Dostálková^1^*, Romana Hadravová^1,2^*, Filip Kaufman^1^, Ivana Křížová^1^, Kryštof Škach^3^, Martin Flegel^3^, Richard Hrabal^4^, Tomáš Ruml^5^ and Michaela Rumlová^1#^

**Supplementary data**

**Supplementary Figure S1**


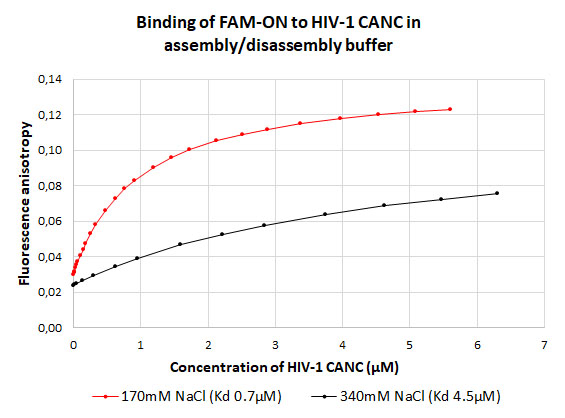


**Supplementary Figure S1. Fluorescence anisotropy measurement**. Dependence of FAM-ON anisotropy on the HIV-1 CANC protein concentration measured in environments with two different NaCl concentrations: 170 mM (red line) and 340 mM (black line). A total of 10 nM of fluorescently labeled 5´FAM ssDNA was titrated with purified HIV-1 CANC protein, and the anisotropy of fluorescence was measured. Kd values for HIV-1 CANC in 170 mM NaCl and 340 mM NaCl were calculated according to the formula described in Materials and Methods.

**Supplementary Figure S2**


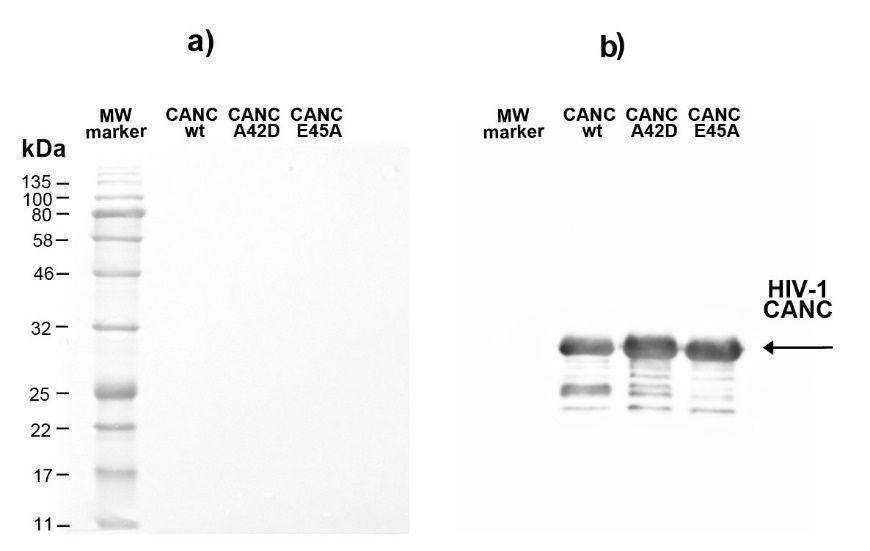


**Supplementary Figure S2. Western blot analysis of wt and mutant HIV-1 CANC proteins.** Purified wt HIV-1 CANC and the A42D and E45A CA mutants were resolved by SDS–PAGE and blotted onto a nitrocellulose membrane. The membrane was incubated with primary polyclonal anti-HIV-1 CA antibody overnight and then incubated with HRP-conjugated secondary antibody for 1–2 h at 4 °C. The antigen–antibody complexes were detected by addition of West Femto Chemiluminescent Substrate (Thermo Scientific, s.r.o., Prague, Czech Republic) and visualized using a FUSION 7S system (Vilber Lourmat, Marne-la-Vallée, France). (**a**) MW marker (Colour prestained protein marker, broad range, NEB) was visualized by a digital camera in bright field. (**b**) HIV-1 CANC proteins were visualized by in chemiluminescent mode using CA by using Fusion CAPT Advance software (Vilber Lourmat, Marne-la-Vallée, France).

**Supplement Figure S3**


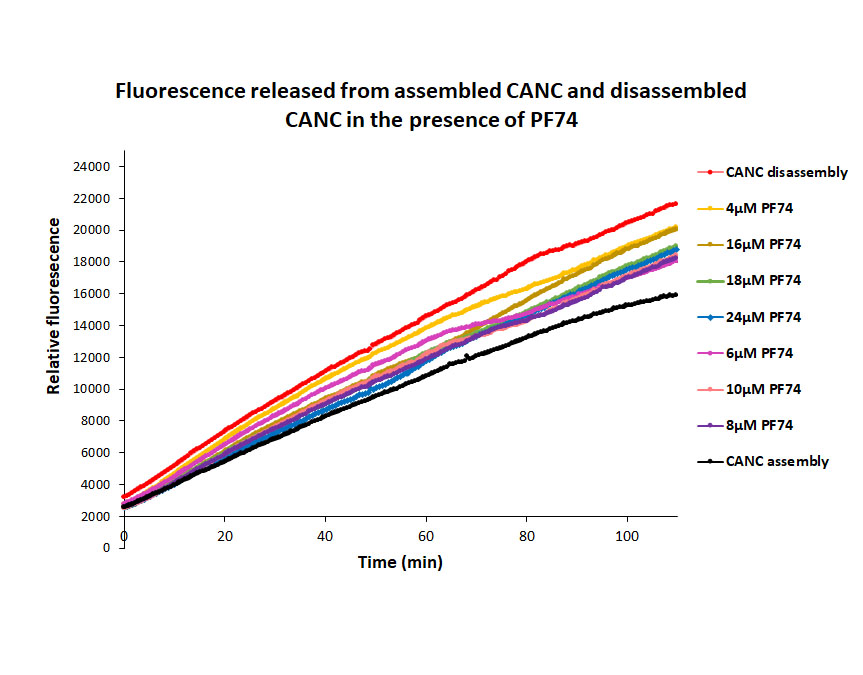


**Supplementary Figure S3. DITH analysis of the effect of PF74 on the stability of HIV-1 CANC.** Tubular HIV-1 CANC particles preassembled in the presence of tqON in assembly buffer were incubated overnight in assembly or disassembly buffer in the absence or presence of indicated concentrations of PF74. Following addition of Exonuclease I, we recorded fluorescence released from CANC assemblies in the absence of PF74 in assembly buffer (black) and disassembly buffer (red), and fluorescence released from the CANC assemblies in disassembly buffer treated with indicated concentration of PF74 shown in yellow (4 µM PF74), pink (6 µM PF74), violet (8 µM PF74), salmon (10 µM PF74), brown (16 µM PF74), green (18 µM PF74) and blue (24 µM PF74). Relative stability of CANC particles was calculated for individual samples.

**Supplementary Table 1: Characterization of PF74**

| **PF74**  *(S*)-*N*-methyl-2-(2-(2-methyl-1*H*-indol-3-yl)acetamido)-*N*,3-diphenylpropanamide  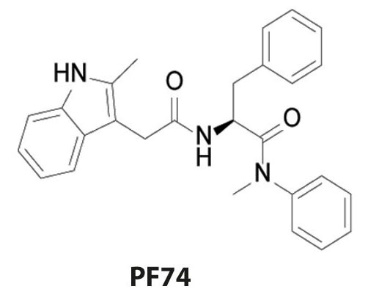 | TLC (hexane:EtOAc, 1:1 v/v): R_f_ = 0.23; ^1^H NMR (400 MHz, CDCl_3_): δ 8.19 (s, 1H), 7.39-7.30 (m, 4H), 7.26 (s, 1H), 7.14 (ddd, J=8.1, 3.3, 1.3 Hz, 3H), 7.11-7.04 (m, 3H), 6.93 (d, J=5.5 Hz, 2H), 6.67 (d, J=7.1 Hz, 2H), 6.22 (d, J=8.3 Hz, 1H), 4.79 (dd, J=15.2, 7.0 Hz, 1H), 3.58 (d, J=3.4 Hz, 2H), 3.17 (s, 3H), 2.73 (dd, J=13.3, 6.9 Hz, 1H), 2.53 (dd, J=13.3, 6.9 Hz, 1H), 2.27 (s, 3H); ^13^C NMR (400 MHz, CDCl_3_): δ 171.23, 170.76, 142.46, 136.06, 135.34, 133.29, 129.73, 129.16, 128.24, 128.08, 127.31, 126.65, 121.46, 119.73, 117.80, 110.41, 104.48, 51.07, 38.70, 37.61, 32.10, 11.55; HRMS/ESI: for C_27_H_28_N_3_O_2_ (M+H^+^) *m/z* 426.21766 found; 426.21760 calculated, for C_27_H_27_N_3_O_2_Na (M+Na^+^) *m/z* 448.19980 found; 448.19955 calculated, for C_27_H_27_N_3_O_2_K (M+K^+^) *m/z* 464.17327 found; 464.17349 calculated. |
| --- | --- |
|  |  |
